# Sex differences in the human brain: a roadmap for more careful analysis and interpretation of a biological reality

**DOI:** 10.1186/s13293-022-00448-w

**Published:** 2022-07-26

**Authors:** Alex R. DeCasien, Elisa Guma, Siyuan Liu, Armin Raznahan

**Affiliations:** Section On Developmental Neurogenomics, National Institute of Mental Health, Bethesda, MD USA

**Keywords:** Sex differences, Neuroanatomy, sMRI, Direct analysis, Meta-analysis, Review, Sex chromosomes, Neurodevelopment, Sexual selection, Anti-sexism

## Abstract

The presence, magnitude, and significance of sex differences in the human brain are hotly debated topics in the scientific community and popular media. This debate is largely fueled by studies containing strong, opposing conclusions: either little to no evidence exists for sex differences in human neuroanatomy, or there are small-to-moderate differences in the size of certain brain regions that are highly reproducible across cohorts (even after controlling for sex differences in average brain size). Our *Commentary* uses the specific comparison between two recent large-scale studies that adopt these opposing views—namely the review by Eliot and colleagues (2021) and the direct analysis of ~ 40k brains by Williams and colleagues (2021)—in an effort to clarify this controversy and provide a framework for conducting this research. First, we review observations that motivate research on sex differences in human neuroanatomy, including potential causes (evolutionary, genetic, and environmental) and effects (epidemiological and clinical evidence for sex-biased brain disorders). We also summarize methodological and empirical support for using structural MRI to investigate such patterns. Next, we outline how researchers focused on sex differences can better specify their study design (e.g., how sex was defined, if and how brain size was adjusted for) and results (by e.g., distinguishing sexual dimorphisms from sex differences). We then compare the different approaches available for studying sex differences across a large number of individuals: direct analysis, meta-analysis, and review. We stress that reviews do not account for methodological differences across studies, and that this variation explains many of the apparent inconsistencies reported throughout recent reviews (including the work by Eliot and colleagues). For instance, we show that amygdala volume is consistently reported as male-biased in studies with sufficient sample sizes and appropriate methods for brain size correction. In fact, comparing the results from multiple large direct analyses highlights small, highly reproducible sex differences in the volume of many brain regions (controlling for brain size). Finally, we describe best practices for the presentation and interpretation of these findings. Care in interpretation is important for all domains of science, but especially so for research on sex differences in the human brain, given the existence of broad societal gender-biases and a history of biological data being used justify sexist ideas. As such, we urge researchers to discuss their results from simultaneously scientific and anti-sexist viewpoints.

**Supplementary Information:**

The online version contains supplementary material available at 10.1186/s13293-022-00448-w.

## Highlights

- Recent large-scale studies have reached different conclusions regarding the presence of sex differences in human neuroanatomy.
- We show that these contradictory findings are explained by different methodological choices. While multiple large direct analyses highlight small, highly reproducible sex differences, reviews do not account for methodological heterogeneity across studies (e.g., statistical power/sample size, brain size-correction methods, segmentation, region selection, participant age). This explains many of the apparent inconsistencies reported in recent reviews.
- We also summarize observations that motivate research on sex differences in human neuroanatomy (including potential causes and effects), review methodological and empirical support for using structural MRI to investigate such patterns, and outline best practices for analyzing and describing neuroanatomical sex differences.
- Finally, we argue that broader historical and societal contexts make it important to reinforce the scientific method by adopting an actively "anti-sexist" viewpoint when conducting research on sex differences in the human brain.

**Supplementary Information:**

The online version contains supplementary material available at 10.1186/s13293-022-00448-w.

## Background

### Introduction

The presence and magnitude of sex differences in human neuroanatomy are hotly debated topics in neuroscience [1–5]. While some studies argue there is little to no evidence for notable, consistent sex differences in human brain structure [1, 6, 7], others report highly reproducible, small-to-moderate sex differences in the size of multiple brain areas, even after controlling for differences in average brain size [2, 3, 8, 9]. With a mounting wealth of data at hand [10], and evermore powerful tools for data analysis [11], how can such contrary beliefs persist around a seemingly simple empirical question? This discrepancy is brought to a head by the titles of two recent papers that spur our current *Commentary*: (1) “*Dump the ‘dimorphism’: Comprehensive synthesis of human brain studies reveals few male–female differences beyond size*” by Eliot and colleagues [1]—a “meta-synthesis” of previously published structural magnetic resonance imaging (sMRI) studies on neuroanatomical sex differences; and (2) “*Sex differences in the brain are not reduced to differences in body size*” by Williams and colleagues [3]—a response to the former article that describes a direct analysis of sMRI data in ~ 40k individuals (aged 40–70 years) [2] from the UK Biobank neuroimaging dataset [12]. Is it possible to reconcile these starkly contrasting conclusions and explain how such conflicting beliefs could emerge?

Our *Commentary* uses the specific comparison between these two recent large-scale studies [1, 3] as a path towards: (i) clarifying the controversy surrounding the topic of sex differences in human neuroanatomy; and (ii) providing a structured framework for the planning, execution, and interpretation of this research. Our goal is to limit miscommunication and promote more effective progress in both scientific and lay discourses on sex differences in human brain anatomy. Throughout this *Commentary*, we make the case that research on sex differences should apply the scientific method in an unwaveringly principled and impartial way, while also actively combating the risks of false extrapolations that draw unfounded conclusions with no support from the data. This approach precludes research that is motivated either by sexism or by the desire to reject the existence of any biological sex differences. There is an undeniable history of biological data being used in attempts to justify sexist views [13–15], and while reporting neuroanatomical sex differences is not an inherently sexist act, modern research on this topic is not isolated from its history. Thankfully, the scientific method offers a clear and objective scaffolding that we can use to help us safely engage with crucial questions [16] regarding potential sex differences in the human brain.

The sections below are organized according to the phases of the scientific method. First, we consider observations that motivate research on sex differences in human brain anatomy. We organize these observations into four groups: (1) concepts from evolutionary theory (i.e., potential distal causes of sex differences in human brain organization); (2) genetic and environmental factors that differ between males and females (i.e., potential mechanistic causes of sex differences in human brain organization); (3) sex differences in the prevalence and presentation of brain-based disorders (i.e., epidemiological and clinical evidence for potential sex differences in human brain organization); and (4) the specific case for using in vivo sMRI as a tool for probing sex differences in human brain organization. Next, we outline best practices for investigations of sex differences in human brain structure, organized according to the following three sequential steps of the scientific method: specifying the question, designing the appropriate study, and framing and interpreting the results. In considering each of these research steps, we highlight how the choices an investigator makes can profoundly impact their final conclusions regarding the existence and significance of sex differences in human brain anatomy. This process helps to make sense of the contrasting results reported by the two recent studies that motivate our *Commentary* [1, 3]. It also offers a more general road map that will hopefully aid effective progress in research on sex differences in the human brain.

In the following sections, we use the term biological “sex” to refer to an individual’s combined sex chromosome complement and gonadal phenotype, which together group most individuals into two categories: males as XY individuals with testes and females as XX individuals with ovaries. Although most individuals can be categorized as female or male based on this system (a designation which is typically made based on genital phenotype at birth), sex is not strictly binary. In fact, intersex individuals, who represent ~ 1% of the population, exhibit an abundance of variation across sex chromosome combinations, sex hormone concentrations, and bodily phenotypes. Bearing in mind this variation, researchers may still meaningfully use biological sex as a concept to examine group-level differences between individuals with "typical" (of the majority) anatomical and sex chromosome combinations, although we acknowledge that these criteria are not confirmed in many of the human studies discussed here, which instead rely on self-identification. Specifically—as for all statistical analyses in biological research—any true group differences should be detectable with sufficient sample sizes, even if there is some amount of intra-group variability or misclassification. Finally, we distinguish biological sex from gender, a culturally defined and malleable concept, and recognize that an individual's gender need not align with their biological sex. Given that an individual's experiences in society can be impacted by their perceived gender, biological and psychosocial influences are often bidirectionally entangled in humans, making their effects difficult to untwine [17]. Amidst this complexity, characterizing phenotypic differences based on biological sex provides one empirically tractable pathway towards better understanding the intertwined influences of sex and gender on the human brain.

## Main text

### Part 1: Observations that motivate research on sex differences in regional brain anatomy

The idea that humans may show sex differences in brain organization is prompted by a wealth of prior theoretical and empirical information from evolutionary, neuroscientific, and medical research. Evolution provides potential distal causes for *why* a phenotypic sex difference may exist (e.g., sex differences in body size as a result of competition over mates), whereas genetic and environmental factors provide potential mechanistic causes that address *how* a sex difference emerges during development (e.g., sex differences in body size being achieved through the sex-biased action of gonadal steroids) [18, 19]. These distinct causal frameworks are complementary (yet sometimes interwoven: [20]), such that attaining an understanding of a mechanistic cause does not preclude the existence of evolutionary explanation [21]. Beyond consideration of causal factors, the high a priori likelihood of human showing sex differences in brain organization is also supported by the existence of very many well-documented sex differences in prevalence, presentation, and prognosis of diverse medical disorders involving the brain. We expand upon each of these motivations for studying sex differences in human brain organization below, before considering why we might expect some of these differences to manifest as sex differences in regional brain anatomy that are resolvable by in vivo sMRI.

#### Potential distal causes of sex differences in human brain organization

150 years ago, Charles Darwin proposed the concept of sexual selection to explain the numerous sex differences (both physiological and behavioral) that he observed throughout the animal kingdom [22]. He outlined two mechanisms, including: (i) mate choice—individuals of sex A who possess a certain version of a trait are more attractive to (and likely to be chosen as a mate by) members of sex B; and (ii) mate competition—individuals of sex A who possess a certain version of a trait can outcompete other members of sex A for mating opportunities with members of sex B. In either case, the individuals exhibiting these trait varieties produce more offspring, leading them to become more prevalent over generations in sex A but not in sex B (to whom these varieties are not beneficial). These mechanisms have not only produced sex differences in visible, physical traits across the animal kingdom—such as the elaborate tails of male peacocks and the large antlers of male deer—but they have also impacted brain evolution. For example, mate choice is responsible for the elegant songs (and larger brain song nuclei) among the males of many songbird species [23], while competition over locating mates has led to greater spatial navigation skills (and larger hippocampi) among the males of certain vole species [24].

Like all other animals, the human species emerged as a result of evolutionary processes that shaped our bodies and behaviors. Although we are distinct from nonhuman animals in many ways (e.g., theory of mind, language, complex tool use) [25], we are not immune to our own evolutionary history, one that certainly included some aspects of sexual selection. For example, when we look across primate species, mate competition tends to be stronger in species with larger sex differences in body size [26]. Accordingly, an evolutionary history involving competition over mates (among males) is among the most viable evolutionary explanations for sex differences in human height and weight [26]. While hypothetical evolutionary causes are hard to empirically assess, sexual selection has provided a powerful explanatory framework for additional sex-biased traits in humans, including facial hair [27], fat distribution [28], aggression [29], and spatial ability [30]. For example: i) mate competition among males may contribute to male-biased rates of aggression across human cultures [29]; ii) sex differences in foraging throughout early human evolution (and after transitions to agriculture in some populations [31]) may partially explain sex-biased spatial abilities (e.g., labor division: females gather from spatially stable but seasonally variable food sites; males hunt across long distances spanning various routes; modern performance patterns: females outperform on object location memory and navigation by landmark tasks; males outperform on mental rotation tasks, which are associated with throwing accuracy and navigation by orientation) [32, 33]; and (iii) effective mate choice may require behavioral inhibition and contribute to female outperformance on inhibitory tasks [34]. If sexual selection did, in fact, shape adult human behavior over evolutionary time, then this will have necessarily been achieved through sex differences in neurodevelopment and adult brain organization. As discussed below, the leading proximal sources of sex-biased brain development are sex differences in chromosomal complement and gonadal type.

#### Potential mechanistic causes of sex differences in human brain organization

The hypothesis that humans may show sex differences in brain organization is not only raised by evolutionary theory, but also by genetic and environmental differences between males and females. Individual differences in sex chromosome complement and the concentration of gonadal sex steroids are foundational to the biological definition of sex in humans and nonhuman mammals, and there is substantial experimental evidence from animal studies that these factors directly impact mammalian brain organization [35]. Accordingly, it is reasonable to expect that human brain development is also subject to direct gonadal and sex chromosome effects. As detailed further below, several lines of observational data support the existence of such effects in humans.

In placental (eutherian) mammals, presence of the *SRY* gene on the Y chromosome of XY males typically leads to differentiation of the embryonic gonadal ridge into testes, whereas the absence of this gene in XX females typically allows differentiation into ovaries [36, 37]. This gonadal divergence tends to lead males and females to exhibit distinct concentration profiles of various sex steroids (e.g., estrogens, androgens) throughout development, although these hormones are not limited to one sex and their concentration distributions overlap [38]. Sex hormones were first shown to shape sex-biased mammalian (rodent) brain organization in the late 1950’s [39], and these findings have been bolstered and extended by the application of a growing suite of experimental methods in murine research [35, 40, 41]. Some of these gonadal influences actually exert experimentally verifiable influences on murine brain anatomy as measured by sMRI [42, 43]. Evidence for similar links between sex steroids and neuroanatomy in humans is largely derived from observational neuroimaging studies that harness non-experimental variations in human sex steroid signaling as a function of: (i) inter-individual variation in circulating sex steroids during development, reflecting variation between individuals in the same developmental period and also across developmental periods (e.g., pre/post puberty, pre/post menopause) [44, 45]; (ii) the menstrual cycle [46]; iii) medical disorders impacting the hypothalamo–pituitary–gonadal axis [47]; or iv) gender-affirming hormone treatment [48].

Although gonadal steroids have been historically viewed as the primary mechanistic drivers of sex differences in mammalian brain organization, there is growing evidence that sex chromosome complement can also have direct effects on mammalian brain organization [49, 50]. For instance, the products of sex chromosome genes are present at different levels in male and female cells [50]: genes on the Y chromosome are expressed only in males and genes that escape X chromosome inactivation (XCI) are often expressed at higher levels females [51, 52]. In addition, XCI in females may partially monopolize cellular epigenetic machinery, altering the expression of other genes [53]. Thus, irrespective of gonadal differences, XY and XX individuals have constitutional differences in the dosage of genes that are known to exert genome-wide regulatory effects that are not mediated by the gonads [54, 55]. This is bolstered by reports that sex-biased expression of autosomal genes in the mouse brain occurs prior to gonadal differentiation [56]. Moreover, there is extensive evidence in both rodents and humans that sex chromosome dosage can influence brain organization [50]. The four core genotype (FCG) model in mice provides experimental evidence that XX and XY groups show several reproducible neuroanatomical differences in sMRI [42, 43], as well differences in vasopressin fiber density within the lateral septum [57]. Evidence for sex chromosome dosage effects on the human brain comes from observational research in groups with differing X and/or Y chromosome doses due to sex chromosome aneuploidy. Specifically, increasing X- and/or Y- chromosome dosage induces highly reproducible changes in diverse aspects of regional brain anatomy, including parieto-occipital cortical surface area (increased), lateral temporal cortical thickness (decreased), and volume of the cerebellum and globus pallidus (both decreased) [58–61].

Alongside the aforementioned effects of gonadal and sex chromosome dosage on the brain, there are also extensively well-documented physiological sex differences in multiple peripheral organs and tissues that may affect the brain. These include adipose [62], hepatic [63], peripheral immune [64], and renal systems [65]. Such somatic sex differences are notable on two grounds: i) they are far less debated or controversial than the existence of sex differences in the brain; and ii) they represent another potential source of sex differences in the brain since they guide concentrations of various peptide and steroid hormones (e.g., leptin, adiponectin, inflammatory cytokines, neurotransmitter precursors, and estrogens) [66].

In addition to the many potential evolutionary, genetic, and endocrine-related causes of sex differences in brain organization, males and females are (at the group-level) often exposed to systematically different environments across the lifespan. For example, the experience of pregnancy has been linked to changes in brain organization [67, 68], and XY males do not experience pregnancy (although fatherhood also impacts the brain: [69]). There is also extensive evidence that individuals who are outwardly perceived as male or female experience pervasive differences in diverse domains of life [70], spanning the educational [71], professional [72, 73], fiscal [74], and medical [75] arenas, in addition to risk of exposure to different dangerous situations [76]. These considerable, consistent, and enduring gendered socio-environmental factors could conceivably influence brain organization in a manner that would manifest as group differences between males and females. It can, however, be extremely difficult (or impossible) to experimentally verify the action of such experiential sex differences on human brain organization, and it is likely there may well be synergy between experientially and biologically driven sex differences. In particular, gendered experiential influences on the human brain may be intimately coupled with sex-biased genetic and endocrine influences [77] since gendered experiences are correlated with an individual’s perceived gender. For example, observed sex differences in aggression and spatial ability may reflect, or be amplified by, gender-biases in the acceptance of and encouragement towards certain types of behaviors and toys throughout development [30, 78].

Taken together, the lines of research reviewed above identify strong evolutionary priors for the existence of sex differences in human brain organization, and also specify numerous endocrine and genetic factors that differ between typically developing human males and females and are known to influence mammalian neurodevelopment. When considering these potential sources of sex differences, however, it is also important to consider how they inform our understanding of the many phenotypic *equivalences* between males and females. At one level, the gonadal and chromosomal differences between typical males and females mean that any features of the mammalian brain that lack a sex difference are necessarily being attained from different starting points in each sex. For example, how do two instances of the same neuronal subtype, one in a male and one in a female, arrive at the same structural and functional profile despite having categorically different sex chromosome gene dosages? Similar questions can be asked about organ-level phenotypic equivalence: how do these two populations, with differences in brain size (which reflect differences in body size), arrive at equivalent intelligence levels [79]? One possible path to phenotypic equivalence between the sexes may actually be mechanistic counterbalancing of two opposing sex differences (i.e., compensatory mechanisms that serve to *prevent* sex differences in brain function and behavior) [80]. For example, experiments in the FCG murine model have identified neuroanatomical regions where sex chromosome and gonadal complement exert opposite effects on volume. These “ying-yang” effects appear to exist in brain areas that exhibit sex-biased volumes (e.g., volume of the medial amygdala is larger in mice with testes than mice with ovaries, but smaller in XY vs. XX mice), as well as regions without any apparent sex differences in mean volume (e.g., the pallidum, where volumetric differences between XX and XY mice only emerge after removal of the gonads, indicating that gonadal effects are “neutralizing” chromosomal effects) [43]. This latter scenario is a specific example of the more generalizable idea that a given phenotype can be similar between males and females but supported by different underlying mechanisms. Additional examples can be found at various biological levels, including interactions between the epigenome and transcriptome (e.g., XCI in females, which results in similar levels of expression for X chromosome genes (that do no escape XCI) in males and females) and between neuroendocrine signaling and behavior (e.g., male and female prairie voles exhibit similar levels of parental care, and while this behavior is produced by pregnancy-related hormonal changes in females, it is driven by vasopressin signaling in males) [80]. Thus, the absence of a sex difference at one level of biological organization cannot be assumed to imply the absence of a sex difference at other levels. Moreover, the preponderance of phenotypic *similarities* between males and females poses fascinating biological questions in light of the aforementioned sex differences in chromosomal dosage and gonadal status.

#### Epidemiological and clinical evidence for potential sex differences in human brain organization

Some of the strongest indirect evidence for biologically driven sex differences in human brain organization is provided by epidemiological and clinical studies that demonstrate large sex differences in the prevalence, presentation, and progression of brain-based disorders [81, 82]. These differences cannot in and of themselves be assumed to solely reflect biologically programmed sex differences in brain organization given the profound gendered influences on health [75], but several observations suggest that biologically grounded factors are likely to be an important contributor. First, multiple neurodevelopmental disorders with onset in early childhood are more prevalent in males than females [81, 83], including autism spectrum disorders (ASD) [84], attention-deficit hyperactivity disorder (ADHD) [85], and early-onset obsessive compulsive disorder (OCD) [86]. In contrast, there is a sharp rise in female-biased risk for depression [87], anxiety [88] and eating disorders [89] in adolescence, and of certain neurodegenerative (e.g., Alzheimer's disease and other dementias) [90] and psychiatric disorders (e.g., late-onset schizophrenia) [91] later in life. The concentrated emergence of sex-biased disorders in windows of particularly dynamic human brain development (i.e., early childhood, adolescent, and post-menopausal years) suggests that sex differences in neurobiology contribute to the emergence of these conditions. Sex biases in sociocultural norms and the applicability of diagnostic criteria are likely to contribute to some of the observed sex biases in disease rates. For example, reduced help-seeking behavior in men may explain their lower treatment rates for depression [92], and current diagnostic guidelines lead clinicians to under-detect ASD in females [93]. However, such biases cannot explain multiple diagnostically distinct disorders all showing the same-sex bias during the same developmental window (e.g., male-biased early-onset ASD, ADHD, and OCD). Second, several sex-biased psychiatric disorders display biological features that interact with known sex differences in neurobiology. For example, the profile of altered gene expression from postmortem brain studies in ASD is partly correlated with normative sex differences in brain gene expression [94]. Similarly, the adolescent-emergent female bias in anxiety is closely linked to measures of pubertal progression that track activation of the hypothalamo–pituitary–ovarian axis in females [88]. Third, medical disorders that modify sex-linked biological factors (e.g., sex chromosome dosage) are associated with increased risk for sex-biased brain disorders [95]. Fourth, males and females exhibit differences in the presentation and progression of multiple neurological conditions. For example: i) females with ASD exhibit more camouflaging behaviors (i.e., compensating for and masking autistic characteristics) and a higher rate of co-occurring internalizing disorders that may mask autistic symptoms, and these factors contribute to the under-diagnosis of ASD in females [93]; ii) mania is more prevalent among males with bipolar disorder, while depression and comorbid panic disorders, eating disorders, and borderline personality disorder are more prevalent among females with bipolar disorder [91]; and iii) males with schizophrenia tend to exhibit higher rates of substance abuse and lower rates of recovery and remission [91]. These differences suggest that sex-specific biological and experiential factors may interact with disease-related factors to impact condition progression. Finally, sex differences in treatment responsiveness continue to emerge. The mechanisms underlying these differences include sex effects on drug metabolism (e.g., in Parkinson’s and schizophrenia treatments) [96, 97] and specific sex–genotype interactions that affect treatment response (e.g., APOE genotype x sex effects on Alzheimer’s treatment outcomes) [98]. Taken together, these diverse sex differences across multiple brain-based disorders provide compelling indirect evidence for sex differences in human brain organization.

#### *The case for probing potential sex differences in human brain organization using *in vivo* structural neuroimaging*

The sections above outline a myriad of evolutionary, genetic, and environmental factors that may produce sex differences in human brain organization, potential evidence for which includes documented sex biases in the prevalence and presentation of multiple brain-based diseases. Taken together, these considerations strongly suggest that humans are likely to show sex differences in brain organization. However, this *Commentary* is specifically focused on the contentious issue of whether humans show sex differences in one particular aspect of brain organization, namely regional brain anatomy as measured by in vivo sMRI [1, 3]. What are the grounds for using sMRI to test for sex differences in human brain organization? This question is fundamental since the human brain offers a vast phenotypic landscape for the manifestation of sex differences, one that spans multiple spatial (from molecules to visible folds) and temporal (from milliseconds to lifespans) scales. We argue that there are both methodological and empirical reasons to test for hypothesized sex differences in human brain organization using sMRI.

From a methodological perspective, in vivo neuroimaging currently offers the only available means of measuring the living human brain in large numbers of individuals. Moreover, in vivo imaging data provide a homogeneous, brain-wide screen for potential sex differences in regional brain anatomy: either to recover effects that have been hypothesized a priori, or to discover novel effects [99, 100]. These properties stand in contrast to postmortem approaches for studying the human brain which, while they enable molecular and microscale measurements, typically require examination of pre-selected regions in small numbers of individuals [50]. Thus, in vivo neuroimaging provides the best available tool to conduct brain-wide tests for sex differences in human brain organization in sample sizes that limit the risk of Type II error (i.e., falsely concluding there is no phenotypic sex difference when one does exist). This is especially true for sMRI, which exists in extremely large datasets [10] and provides a vast array of different structural brain features (e.g., cortical thickness, area, folding, etc.) that can be similarly measured with high reliability across humans of diverse developmental stages (e.g., in contrast to task-based functional neuroimaging) and across humans and nonhuman animal models [11].

Fortunately, beyond the methodological “pushes” to test for sex differences in human brain organization using sMRI, there are also multiple empirical “pulls” for positing that sex differences in mammalian brain organization could manifest in ways that are detectable by sMRI. First, in species like mice, where both postmortem and in/ex vivo sMRI data are available, sMRI is clearly able to reliably recover all classical histological foci of sex-biased regional brain volume, and also discover new foci that have been missed by the regionally targeted nature of most postmortem research [35, 50, 100]. Second, newer tools for brain-wide analyses of cell counts in mice show that many foci with sex-biased cellular compositions are also regions that show sex-biased volumes by sMRI [100, 101]. Third, gonadal and sex chromosome factors that tend to differ between typical males and females have been shown to modulate sMRI measures of regional brain volume using experimental methods in mice [42, 43] and observational data in humans [47, 48, 58–61].

### Part 2: Specifying the question: do humans show sex differences in regional brain anatomy by sMRI?

The aforementioned observations strongly motivate empirical tests for regional sex differences in human brain volume using in vivo sMRI. However, conducting such a test requires first specifying what one means by “sex” and a “sex difference”. That is, we need to properly define the groups being compared, and the observations that would constitute a group difference. When testing for sex differences in regional brain volume, it is also necessary to specify if and how any adjustment will be made for well-established average sex differences in height and total brain volume. We consider each of these issues below.

#### Specifying terms of reference when referring to “sex”

The need to define terms of reference in formulating a research question is particularly relevant for the study of sex differences. Even the apparently straightforward notion of biological sex is not a clear binary: sex chromosomes are not sex specific, sex hormones are not sex limited (i.e., androgens and estrogens are not male- and female-specific, respectively), and sex presentation does not perfectly reflect circulating hormones or chromosome complement [102]. Accordingly, when testing for sex differences, it is important to explicitly state how sex is being defined. Definitions may include self-reported sex or karyotypic sex.

#### Specifying terms of reference when referring to a “sex difference”

It is also important to clarify the terminology being used to describe the type of sex difference being considered. Here, we present a slightly modified version of the framework outlined by McCarthy and colleagues [35]: (1a) “sexual dimorphism” should only be used when a trait has two forms, one more prevalent in males and one more prevalent in females (usually limited to traits directly associated with reproduction); (1b) “sexual polymorphism” should be used when a trait has more than two forms (compared to sexual dimorphism, this term more accurately reflects variation from e.g., sex chromosome aneuploidies, intersex physical phenotypes) [103]; (2) “sex difference” should be used when the trait exists on a continuum, with males and females exhibiting mean or variability differences; and (3) “sex convergence and divergence” should be used when the trait does not exhibit a sex difference but comprised different neural underpinnings. In addition, a useful alternative may be to describe “sex differences” as the result of “sex influences” [104], since this term may help prevent incorrect assumptions of large categorical differences. Clarity in terminology is not only important for accurately conveying one’s own research findings to others, but also for properly interpreting published research and discussing perceived conflicts or controversies in research. The conflation between “sexual dimorphisms” and “sex differences” is particularly problematic, and can lead well-intending researchers to erroneously describe potential sex differences as sexual dimorphisms (e.g., [105, 106]), or readers to mistakenly conclude that reported sex differences are being taken by their authors to imply evidence for a sexual dimorphism [1].

#### Adjustments for height or total brain size

Another critical clarification when studying sex differences in human neuroanatomy relates to the facts that: (1) average body size and overall brain volume are larger in males than in females [3, 107]; and (2) regional volumes show strong positive correlations with overall brain volume that are region-specific in their magnitude [108]. These observations mean that all absolute regional brain volumes are, on average, larger in males than females, and that certain brain areas tend to comprise a larger or smaller proportion of male versus female brains, depending on whether those areas scale hyper- or hypo-allometrically with brain size, respectively [59, 108–111]. Accordingly, many studies seek to control for sex differences in overall brain size when investigating sex differences in regional anatomy. The neurobiological rationale for focusing on sex differences in regional brain volume stems from the partly localized nature of structure–function mappings in the mammalian brain [112, 113], which imply that any domain-specific functional sex differences (e.g., of reproductive or social behavior) are more likely to be related to network-specific (versus global) sex differences in brain organization. Additionally, there is evidence that species differences in relative, but not absolute, brain region size correlate with species differences in behavior and cognition (e.g., relatively, but not absolutely, larger hippocampus volumes are observed in food caching vs. non-caching bird species) [24]. In specifying one’s question regarding the existence of sex differences in regional brain anatomy by sMRI, it is not just important to state *if* brain size is being controlled for, but *how* any control is implemented. This is a critical issue because some popular methods for controlling brain size in analyses of group differences induce predictable biases in analyses of neuroanatomical sex differences (see ‘Designing the appropriate study’ below).

The above considerations lead many contemporary analyses of sex differences in the human brain to be guided by a very specific question: “Do self-identified men and women exhibit statistically significant average differences in brain region anatomy, as detectible by in vivo sMRI, after controlling for differences in overall brain size?” This is the question considered by both of the recently published articles that prompted the current *Commentary* [1, 3]. Although both studies considered the same question, and drew on large bodies of data in search of an answer, they arrived at diametrically opposed answers. We show below that this strange state of affairs is a predictable consequence of differences in study design, and we argue that clarity around the impact of different study design choices is a critical ingredient for rational progress on the question of sex differences in human brain anatomy.

### Part 3: Designing the appropriate study to test for sex differences n regional human brain anatomy based on sMRI data

A theoretically definitive study design to examine sex differences in regional human brain anatomy based on sMRI data would be to: (i) use the same MRI machine to gather structural brain scans on all male and female humans (defined by e.g., karyotype and circulating sex hormone concentrations) in a target population of interest ascertained using epidemiological sampling principles (e.g., minimizing sampling biases) [114]; (ii) preprocess all scans using an identical analytic pipeline [115]; (iii) use statistical methods to estimate sex differences in regional brain anatomy after considering sex differences in neuroanatomical variability [116, 117], variables that can influence regional brain anatomy (including total brain volume and age), and potential multicollinearity between variables. This study design is impractical, but provides a theoretical ideal against which alternative study designs can be assessed. To date, research on sex differences in regional brain volume has used one of two main study designs, which are considered in turn below.

#### Approach 1: direct analysis of sex differences in regional brain anatomy using large sMRI datasets

To date, most researchers studying sex differences in human brain anatomy by sMRI have sought to approximate the idealized study design above by analyzing a subsample of the population to estimate true effects in the full population. This study design is exemplified by the recent studies from Williams and colleagues [2, 3], which provide a direct analysis of regional sex differences in brain volume using the largest single-study dataset available to date: ~ 40,000 sMRI scans from the UK Biobank dataset [12]. These papers concluded that males and females showed statistically significant differences in 67% (409/620) of all cerebral measures analyzed, even after controlling for brain size, age, and technical effects. Given that the vast sample size of this study is likely to facilitate small group differences reaching statistical significance, the authors quantified the distribution of effect sizes (i.e., standardized betas) for statistically significant group differences, which ranged from -0.67 to 0.64 (median absolute effect size = 0.13). While most differences were “small” (less than 0.1), 46% of regions had an absolute effect size greater than 0.1 (corresponding to a sex difference in region volume equal to 10% of the standard deviation of log-transformed region volume, holding all other factors equal). For example, the reported standardized sex effect for right hippocampal volume was ~ 0.1 (0.098), which corresponds to females having ~ 20mm^3^ (or ~ 1.5% of the average) larger volumes compared to males with same brain size, of the same age, and whose data were collected under the same technical parameters.

The work by Williams and colleagues [2, 3] demonstrates many of the advantages in testing for sex differences through direct analysis of large sMRI datasets: (i) there is adequate statistical power to confidently detect even small effect sizes; (ii) the effect sizes can be accurately estimated, enabling researchers to go beyond statistical significance and convey the magnitude of observed group differences; and (iii) exquisite analytic control allows investigators to systematically test if and how the statistical significance and effect size of any observed sex differences are altered by consideration of co-occurring technical, age, or brain size effects. Williams and colleagues pay close attention to the issue of brain size control by first systematically mapping non-linear scaling relationships across the human brain, showing that these scaling laws are almost always identical between males and females (with no sex difference for 99.8% of regions), and testing for sex differences in regional brain anatomy using a log–log regression method that avoids the biased effects caused by other brain size adjustment methods [2] (discussed more below). Notably, the work by Williams and colleagues was able to directly test for such complex phenomena (e.g., potential age modulation of scaling effects) and to, therefore, properly account for any such effects (which were found to be minimal in this study [2] and other large-scale reports [111]).

**Fig. 1:**
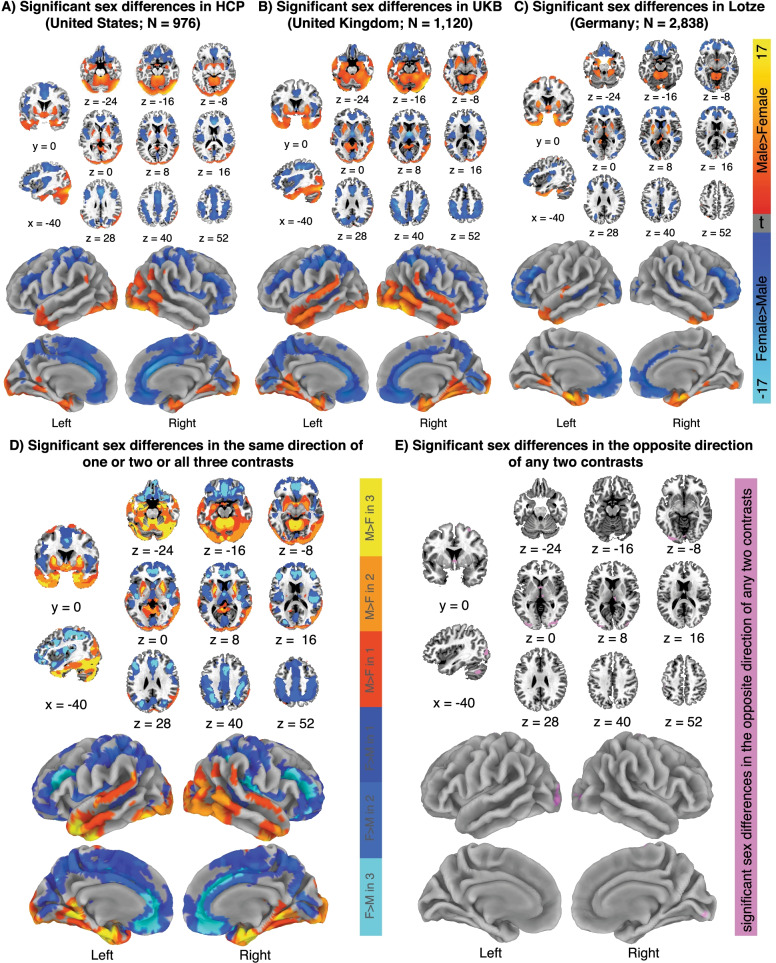
The spatial patterning of neuroanatomical sex differences is largely reproducible across 3 large cohorts. Sex differences in regional gray matter volume (after brain size-correction) across 3 independent cohorts. **A** Results from the HCP cohort (*N* = 976) analyzed by Liu and colleagues [8]. **B** Results from the UKB subsample (*N* = 1120) analyzed by Liu and colleagues [8]. **C** Results from the SHIP cohort (*N* = 2838) analyzed by Lotze and colleagues [9]. **D** Conjunction map across 3 cohorts. Color encodes whether 1, 2 or 3 of these cohorts show overlapping statistically significant sex differences in regional GMV (cool colors, F > M, warm colors: M > F). Note the spatial nesting of colors (this is consistent with a core pattern of sex biases in regional GMV that is variably recovered by these three studies). **E** Map of inconsistencies across cohorts. Purple regions are those where any 2 of the 3 cohorts considered showed statistically significant regional GMV sex differences in opposite directions

An additional advantage of studying sex differences in human neuroanatomy via direct data analysis was highlighted by two other recent MRI studies [8, 9] which harnessed their large sample sizes to provide direct quantitative tests of the reproducibility of sex differences across different cohorts [8, 9]**.** Lotze and colleagues [9] examined two independent cohorts from the Study of Health in Pomerania (SHIP) (N ~ 1000 and N ~ 1800) and found that all regions exhibiting significant sex effects in the smaller cohort were reproduced in the larger cohort. Similarly, Liu and colleagues [8] found that the spatial distribution of sex differences in GMV was highly reproducible across: (i) two large cohorts (N ~ 1000 each) drawn from the Human Connectome Project (HCP) and the UK Biobank (UKB) (r > 0.80); and ii) 1000 split-half samples of the HCP dataset (mean r ~ 0.8). Accordingly, these studies demonstrate the reproducibility of sex differences in regional human neuroanatomy by directly comparing estimated sex differences in different samples and quantifying the reproducibility of the map of anatomical sex differences using spatial correlations both within and between samples. Importantly, Liu and colleagues [8] directly quantified the within-cohort reproducibility of sex differences using permutation methods that can accommodate data distributions that violate some assumptions of parametric methods (e.g., the assumption of equal variances in linear regression between males and females). Such focused comparisons of large datasets represent the gold standard approach to reproducibility since, compared to meta-analyses of many methodologically heterogeneous studies (discussed below), they facilitate more straightforward analysis and interpretation. Figure 1 reproduces and directly compares the spatial patterning of significant sex differences in regional gray matter volume (after brain size correction) across the 3 independent samples, including the SHIP cohort (Fig. 1A), HCP cohort (Fig. 1B) and UKB subsample (Fig. 1C). A conjunction of these 3 maps (Fig. 1D) demonstrates that humans show a highly reproducible spatial pattern of sex differences in regional GMV, including male-biased volumes of the putamen, amygdala, hippocampus, and temporal pole and female-biased volumes of the cingulate, superior parietal, and lateral prefrontal cortices. Moreover, this conjunction analysis reveals spatially nested levels of consistency, pointing to steady mounting levels of cross-study agreement approaching specific anatomical foci (Additional file 1: Table S1). Our conjunction also helps to specify the small number of foci with opposing directions of statistically significant sex differences across the 3 studies (Fig. 1E, Additional file 2: Table S2). We consider how to better understand these inconsistencies below.

#### Approach 2: re-analysis of previously published studies of sex differences in regional human brain anatomy

Prior to the availability of large sMRI datasets like the UK Biobank [12], the only way to probe potential sex differences in human neuroanatomy while achieving relatively large sample sizes was to combine the results of previously published studies. However, re-analyses of prior publications suffer from several key limitations as compared to direct analysis of large datasets. Whereas direct data analysis in large samples can provide precise estimates of the effect size for a given sex difference in regional brain anatomy under pre-specified methodological conditions (e.g., methods for segmentation or controlling for brain size), re-analyses of prior publications have to combine findings across diverse studies that vary in sample size and methodology, and are likely to be affected by publication bias towards positive findings [120]. The best way of mitigating sources of heterogeneity and testing for publication bias in review is through meta-analysis, a statistical process for combining data from multiple studies after weighing evidence by sample size to derive summary effect size estimates [6, 7, 121]. However, reliable and interpretable summary effect sizes for neuroanatomical sex differences can only be computed by meta-analysis when there are multiple primary studies that share common approaches to anatomical measurement and statistical control for brain size variation. Unfortunately, the methodological heterogeneity of published sMRI studies on sex differences has precluded meta-analysis across all major study design permutations.

In contrast to the meta-analytic approach, studies that seek to summarize past reports without formal meta-analysis are referred to as “systematic reviews” if they explicitly define literature search terms and study inclusion/exclusion criteria [122] or “reviews” if they do neither of these things. The “meta-synthesis” by Eliot and colleagues [1] is a review that tallies past reports according to whether they found a given anatomical measure to be significantly larger in males, larger in females, or not significantly different between the sexes.

Whether by meta-analysis, systematic review, or review, any attempt to assess sex differences in regional human brain anatomy by combining results of prior research should weight the available evidence by two key study design considerations. First, it is critical to weigh the evidence from past studies by their sample size. This procedure is necessary because of the basic statistical fact that larger sample sizes drawn from a target population provide more accurate and stable estimates of parameters (e.g., the true population mean). By contrast, small sample sizes may produce spurious results or exaggerate true effect sizes [2]. Second, it is crucial to stratify prior studies by the approach taken for modeling brain size variation. This is because some common techniques for brain size-correction introduce directional biases into the estimation of neuroanatomical sex differences, due to interactions between sex differences in average brain size and the existence of region-specific, non-linear relationships between region volumes and total brain volume [59, 109–111]. For example, the proportion method (i.e., taking a ratio of region size to overall brain size) assumes that all regions take up the same proportion of the brain regardless of brain size (i.e., ‘isometry’). Accordingly, for regions that scale hyper-allometrically with brain size (i.e., take up a larger proportion of larger brains) (e.g., cerebral white matter), proportionalization will artificially decrease region size in females and increase region size in males. Conversely, for regions that scale hypo-allometrically with brain size (i.e., take up a smaller proportion of larger brains) (e.g., amygdala), proportionalization will artificially increase region size in females and decrease region size in males. Additionally, the residual adjustment method (i.e., using the residuals from a regression of region size on brain size) can lead to biased results when brain size is correlated with any of the other predictors (e.g., sex). These biases can create or obscure sex differences in brain anatomy. Such distortions are substantially reduced, or avoided completely, by studies that include brain size as a covariate in multiple regression models. In particular, given that the volume of many brain regions scales non-linearly with brain size, it is recommended to log-transform region and total brain volumes [2]. This transformation is necessary to induce a linear relationship between region volume and overall brain size, and can affect results (see e.g., Williams and colleagues’ [2] for comparisons of linear and non-linear adjustment methods).

Thus, tallying the results of published studies without considering the key methodological factors detailed above would be predicted to suggest highly inconsistent findings regarding sex differences in human brain anatomy, which is precisely what is found in the review by Eliot and colleagues [1].

#### Consideration of scientific study design is crucial when weighing published studies on sex differences in human neuroanatomy

Based on their review of past studies, which collectively included up to ~ 30k participants, Eliot and colleagues conclude that “*once we account for individual differences in brain size, there is almost no difference in the volume of specific cortical or subcortical structures between men and women*” [1]. In contrast, based on their direct analysis of ~ 40k brain scans, Williams and colleagues conclude that “*we find that sex differences in total brain volume are not accounted for by sex differences in height and weight, and that once global brain size is taken into account, there remain numerous regional sex differences in both directions*” [3]. The study design considerations reviewed above can help explain this discrepancy.

**Fig. 2:**
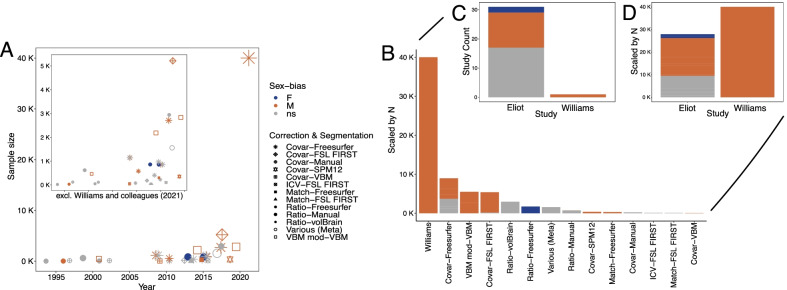
Methodological differences across studies explain apparent inconsistencies in reported sex differences in amygdala volume. Results and design characteristics of studies on sex differences in amygdala volume, including the direct analyses by Williams and colleagues [2] and the studies collated by Eliot and colleagues [1] (*N* = 31). **A** Each point represents one study: color = detected sex-bias, size = sample size, shape = brain size correction and segmentation method combination (see legend). Inset depicts the plot excluding Williams and colleagues [2]. **B** Bar plot depicting the sex-bias (color) per correction and segmentation method (with the Williams et al. study isolated), scaled by the sum of the underlying study sample sizes. **C** Bar plot depicting the sex-bias (color) per correction and segmentation method, scaled by the underlying study counts. **D** Bar plot depicting the sex-bias (color) across the studies tallied by Eliot and colleagues and the study by Williams and colleagues, scaled by the sum of the underlying study sample sizes. Loss of information regarding analytical methods and sample sizes accounts for apparently inconsistent findings. Notably, the only studies that detect female-biased amygdala volumes use the proportionalization method for brain size correction (which introduces biases—see text) combined with Freesurfer segmentation. Studies that report non-significant differences tend to have smaller sample sizes (mean N across studies: *ns* *N* = 557; female-biased: *N* = 859; male-biased: *N* = 4366) or are meta-analyses of studies that used various methods. In fact, all large studies that used the covariate or VBM correction methods (*N* = 6 studies with sample sizes > 1000) detected male-biased amygdala volumes in their primary analyses

The overlapping regional sex differences in brain volume across 3 independent samples (Fig. 1, Additional file 1: Table S1 [8, 9],) and the high statistical precision with which Williams and colleagues were able to detect regional anatomical sex differences in the full 40k UKB sample [2] suggest that methodological factors may have led Eliot and colleagues’ literature review to underestimate the consistency of sex differences in regional brain anatomy. This hypothesis is supported by the observations that: i) individual reports collated by Eliot and colleagues varied greatly in their sample sizes and techniques for anatomical analysis; and ii) results of these studies were tallied without adequate adjustment for these study design features. The problem posed by this heterogeneity is well-illustrated by considering Elliot and colleagues’ results tally for the amygdala [1]—a region that consistently demonstrates significant male-biased volume (in the left or both hemispheres) across multiple recent large-scale studies [2, 8, 9] (Fig. 1, Additional file 1: Table S1), as well as in the study by Williams and colleagues [2]. Figure 2A shows the individual studies of amygdala volume that were tallied by Elliot and colleagues, plotted as a function of date, sample size, and technique for brain size control, with the study by Williams and colleagues included for reference. This plot highlights the gross heterogeneity across these study properties, the sharp recent rise in sample size yielding studies with similar findings, and the order-of-magnitude increase in sample size represented by William and colleagues’ recent study. Figure 2C provides a direct visualization of the Eliot and colleagues’ tally of past findings for regional amygdala volume, while Fig. 2B and D re-visualizes this tally with additional information on study sample size and methodology. We also provide a sample size weighted entry for the results of Williams and colleagues [2]. This sample size and methodology-informed re-tallying of the studies reviewed by Eliot and colleagues reveals a strong signal from past literature for mean amygdala volume being larger in males than females after accounting for brain volume using unbiased methods, which is precisely the result reported by direct MRI data analysis in multiple recent large studies including Williams et al. [2, 8, 9].

We provide the above example to illustrate the inherent limitations in using review or meta-analysis across multiple small samples to draw conclusions regarding the existence and reproducibility of sex differences in regional brain anatomy [1]. Many of these issues can be avoided in direct analyses of the large and well-powered neuroimaging datasets (Fig. 1, Additional file 1: Table S1, [2, 8, 9]) that we are now so fortunate to have access to [10]. Careful attention to study design not only helps to resolve apparent inconsistencies throughout past research on sex differences in human neuroanatomy, but also provides an objective, rational framework to help researchers and general readers sensibly weigh any future research on this topic.

#### Designing studies to understand inconsistent results

Despite the overwhelming consistency across large, well-conducted studies of sex differences in human neuroanatomy (Fig. 1D, Additional file 1: Table S1), there are also some inconsistencies (e.g., Fig. 1E, Additional file 2: Table S2). For example, at the granular voxel-level, we find that 2 or more cohorts examined by the recent large-scale studies of regional sex differences in GMV find opposing statistically significant sex differences for the calcarine sulcus and multiple cerebellar areas (Fig. 1E, Additional file 2: Table S2). These inconsistencies are few but represent opportunities for us to improve our understanding of neuroimaging research on neuroanatomical sex differences in humans. Specifically, targeted research can assess whether a lack of reproducibility in these specific regions reflects particular statistical, technical, or biological factors. Previous studies have highlighted multiple factors that are likely to be critical, including: (i) differences in statistical power due to sample size variation; (ii) the use of different brain size correction methods; (iii) the use of different segmentation approaches; (iv) the use of voxel-wise versus region-wise approaches; (v) region-specific sex differences in age-related size changes; and (vi) subregion-specific sex differences. For instance, Williams and colleagues [2] replicated a previous analysis of a subsample of the UKB (N ~ 5200) [123] by applying the log–log regression method (versus the linear covariate adjustment) and an alternative subcortical segmentation approach. Although a majority of the sex effects were replicated, the replication analysis detected sex differences for multiple regions that were not identified in the original study. Similarly, Williams and colleagues [2] found that different cerebellar segmentation approaches produced opposite results (i.e., cerebellar GMV was female-biased in one analysis and male-biased in another), a finding that highlights potential issues with automated (versus manual) segmentation approaches required by studies with extremely large sample sizes. Variation across segmentation methods may be particularly relevant to structurally complex regions. Furthermore, the use of voxel-wise versus region-wise approaches can lead to discrepancies across studies, since the former may detect sex differences within *part* of a region, but when the sex-biased area does not comprise a large enough portion of the overall region size, no difference is detected by the latter (e.g., the right amygdala is male-biased according to voxel-wise comparisons in [8, 9] but exhibits no bias under region-wise comparisons in [2]). We might also expect to find inconsistent results across studies of different age groups when there are region-specific interactions between sex and age (linear or quadratic) [2, 9]. While such interactions may be relevant to a number of brain areas [2] this issue may be particularly relevant to the hippocampus, a developmentally plastic structure in which adult neurogenesis occurs [124]. Analyses of this region also suggest that sex-by-age interactions vary across different subregions of the hippocampus [2, 9], suggesting that seemingly inconsistent results for functionally and structurally heterogeneous structures (like the hippocampus) may be explained by examining subregion-specific sex differences. Similarly, it would be more appropriate to analyze a region like the hypothalamus on a subregion level, as it is composed of numerous functionally distinct nuclei that are likely to differ in their patterns of sex-biased volume in humans (as in mice [125]). Thus, when large, well-conducted studies generate inconsistent findings regarding sex differences in regional brain anatomy, these inconsistencies should themselves be treated as an object of scientific inquiry to understand the biological or methodological factors that might drive heterogeneous results. Moreover, while seeking to understand inconsistencies, special scrutiny should be applied to results that originate from studies that have smaller samples and use suboptimal methods for brain size control.

### Part 4: Framing and interpreting the results

Confusion and ineffective communication around the topic of sex differences in human neuroanatomy is not only shaped by the decisions made en route to generating results, but also by the way these results are discussed and interpreted. Below, we consider important issues in the interpretation of reported sex differences in regional human brain anatomy.

#### Exploratory versus hypothesis-driven approaches to the study of neuroanatomical sex differences

The observations outlined earlier in this *Commentary* (e.g., sexual selection mechanisms, environmental sex differences, the utility of in vivo sMRI) represent broad hypotheses and assumptions that may guide us towards the exploratory approaches most likely to improve our understanding of human brain sex differences [126]. However, many of these observations do not represent the kinds of specific hypotheses envisioned by most hypothesis-driven accounts of scientific method [126]. The relative value of exploratory versus hypothesis-driven approaches has been debated for decades, and although more targeted analyses (of specific areas) with pre-registered research plans prevent researchers from making post hoc rationalizations for sex differences detected from whole brain screens [17], modern data-intensive scientific disciplines do tend to be characterized by an iterative interplay between hypothesis-driven and exploratory research [126]. This iterative model of scientific practice suggests that any study design can be appropriate if it incorporates approaches and methods that best address gaps in current knowledge, even if the research does not focus on hypothesis testing [126]. While this viewpoint bolsters the utility of exploratory studies of neuroanatomical sex differences, it also highlights the value and complementarity of targeted, hypothesis-driven studies. Researchers implementing either approach should aim to prevent misinterpretations of their findings by explicitly stating the exploratory nature of the work upfront (with appropriate statistical correction for multiple comparisons) and avoiding speculative, functional extrapolations for which there is no evidence (discussed more below).

#### The complex relationship between sex and gender and the need for intersectional approaches

Studies examining potential sex differences in human brain anatomy should not only be clear regarding their definition of sex (see above), but also recognize the distinct concepts of sex versus gender. Indeed, more studies are explicitly considering participants’ sex *and* their gender to more accurately represent individuals on both spectra and recognize the likelihood of separate, compounding, and interacting effects of these variables on disease susceptibility, presentation, and diagnosis [127–129]. Although this approach is still in its infancy, it is likely to represent a critical step towards understanding the entwined social and biological drivers of these conditions. Furthermore, intersectional approaches that consider potential interacting effects of other social identities and categories (e.g., ethnicity) are critical since gender effects can only be fully understood in the context of these identities.

#### Identifying sex differences versus classifying individuals by sex

The studies discussed above largely focus on the specific question: Do humans show sex differences in regional brain anatomy by sMRI? This is distinct from the multivariate classification version of this question: Can anatomical variation (captured by e.g., sMRI-derived measures) across multiple brain regions be used to reliably classify the sex of an individual brain? Many studies have demonstrated that multivariate classification algorithms can, in fact, predict sex based on neuroanatomical features with good accuracy [4, 130]. However, this approach can limit our understanding of individual variation within the sexes [5, 131]. Furthermore, these findings should not be interpreted as evidence for large sex differences in brain structure since classification and estimation are very different concepts [132]. Accordingly, identifying specific brain areas that exhibit sex-biased volumes, estimating the magnitude of those average differences, and investigating inter-regional and inter-individual deviations from these averages requires the approach used in many studies discussed here (including those by Williams and colleagues [2, 3]), namely direct analysis of sex differences in regional brain anatomy using large sMRI datasets.

#### Expectations and interpretations of the magnitude of neuroanatomical sex differences

The increasingly large sample sizes available for studies of human neuroanatomy make it possible to recover statistically significant sex differences in regional brain anatomy that involve small sex differences in mean volume [133]. The small magnitude of most neuroanatomical sex differences is sometimes used to dismiss these differences as unimportant [1], but whether the size of these differences falls below, exceeds, or is in line with prior expectations has rarely been addressed in studies of neuroanatomical sex differences.

**Fig. 3:**
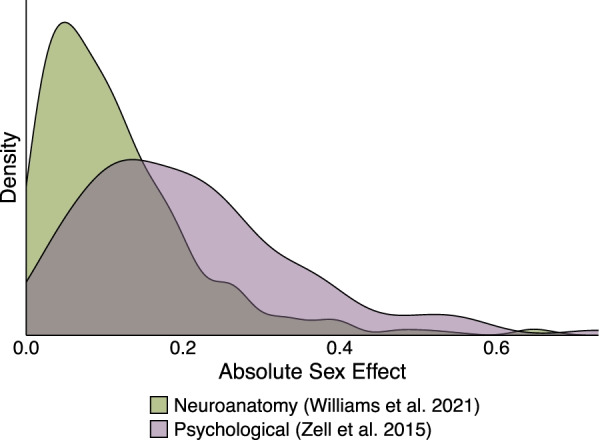
Effect size ranges for sex differences in neuroanatomy and psychological measures. Density plot of absolute sex effects (either Cohen’s d or standardized betas) on neuroanatomical and psychological variables. Neuroanatomical sex effects are depicted for 620 neuroanatomical volumes, surface areas, and thicknesses from Williams and colleagues (measured in [2], depicted in [3]). Psychological sex effects were derived from 106 meta-analyses (collated in [137]) across multiple domains, including cognitive (*N* = 30), personality/social (*N* = 65), and well-being (N = 11)

Comparative biology can provide some insights that may guide these expectations. Previous work suggests that species with smaller sex differences in behavior and environment also tend to exhibit smaller brain sex differences [23, 24, 134, 135]. For instance, songbird species with male-limited singing show large sex differences in the size of brain song nuclei, while species with male–female duetting tend to exhibit much smaller differences [23]. Similarly, monogamous vole species exhibit small sex differences in home range size, spatial ability, and relative hippocampus size, but these measures are male-biased in polygynous species since only males need to search for mates [24]. Given that humans exhibit little to no sex differences for many cognitive and psychological traits [136, 137], it follows that we should expect any structural sex differences in the human brain to *also be small* (Fig. 3). Given this expectation of small average sex differences, it also follows that we *should not* expect non-overlapping distributions for neuroanatomical measures between the sexes (i.e., sexual dimorphism). Notably, an observation of overlapping distributions does not reject a hypothesis centered on group-level mean differences. This is consistent across countless non-sex-related study designs created to test whether there is a significant effect of a binary trait (e.g., the Western versus Mediterranean diet) on another, continuous trait (e.g., BMI).

Nevertheless, neuroanatomical sex differences should not simply be dismissed because they are “small”. In fact, previous work suggests that integrating multiple individual measures (each with small effect sizes) into a multivariate measure can produce a much larger effect size that implies statistical separation of males and females (e.g., sex differences in some personality traits and aggression) [138, 139]. Furthermore, there is no a priori reason to assume that large, widespread structural differences are required to produce the observed sex differences in disease prevalence and presentation (discussed above). The magnitude of biological and/or functional implications of empirically small anatomical differences cannot be assumed based on the latter. For example, duplication of the Y chromosome represents an empirically small amount of excess genomic material (chrY =  ~ 2% of the genome), but it can result in large neuroanatomical and behavioral alterations (e.g., ~ 18% increase in ASD prevalence; ~ 67% increase in special educational needs; both vs. national prevalence rates in England) [140]. Similarly, small volumetric differences may reflect empirically larger differences in finer aspects of neurobiology (e.g., cell counts) [101] that may be relevant to disease susceptibility. Accordingly, *small average* sex differences in brain structure may potentially help to illuminate the mechanisms underlying differences in disease, either directly or by revealing regional sex differences in other levels of biological organization that are proxied by anatomy.

#### Causes and effects of neuroanatomical sex differences

Studies that describe a sex difference in average region volume do not provide any information regarding the causal bases for that difference. In particular, these studies cannot illuminate which microstructural features underpin the observed anatomical differences, which proximal genetic and/or environmental factors may establish these differences, or which (if any) distal evolutionary factors were at play throughout early human evolution. Rather, as detailed above, the value of sMRI studies is that they help to prioritize brain systems for closer investigation using other selected research approaches.

Previous work suggests that structural sex differences may reflect underlying differences that cannot be directly inferred from volume and that may even exhibit the opposite-sex bias [101]. Accordingly, sMRI-directed postmortem techniques would be required to study the microstructural bases for in vivo differences in regional brain volume in humans. In contrast, experimental animal studies would be needed to probe potential mechanistic drivers of sex-biased brain development [42, 141], with the attendant caveats that arise when using certain model systems to study human phenotypes [142, 143]. Such research would also need to explicitly grapple with the multi-level nature of brain organization, the possibility that sex differences may show a complex relationship between different levels of biological analysis, and the fact that many detected sex differences are likely to represent compensatory mechanisms that facilitate equal functionality across individuals [80]. Finally, an understanding of the evolutionary drivers of neuroanatomical sex differences in humans may never be fully within our grasp, but there is potential for comparative studies to provide some insights (for non-primate examples, see: [134, 144]). This approach will require much larger sex-specific neuroanatomical (e.g., sMRI-derived) and behavioral data sets (both within and across primate species) than are currently available.

Furthermore, the act of simply describing a sex difference in regional brain anatomy is entirely distinct from understanding the functional implications (if any) of such a difference. This not only reflects a lack of understanding of the neurobiological drivers of regional volumetric differences (see above), but the wider complexity of structural and functional relationships between brain regions, as well as how such relationships relate to inter-individual differences in behavior or cognition. While published reports of links between neuroimaging sex differences and sex differences in cognitive or behavioral traits do exist (reviewed in [145]), the neuroimaging community is currently grappling with difficult general questions regarding the degree to which in vivo structural and functional features from MRI can predict inter-individual variation in human behavior or cognition [146]. In studies of neuroanatomical sex differences, these questions are compounded further since there are many diverse configurations that could theoretically underlie sex differences in brain–behavior relationships. For example, given a hypothetical dimension of behavior that correlates with neuroanatomical variation within a network of brain regions, males and females may differ in: (i) the set of brain regions that constitute this network; (ii) the strength of brain–behavior correlations for individual regions; (iii) the relationship between behavior and anatomy across multiple regions; and (iv) the specific anatomical feature (e.g., cortical volume vs. cortical thickness) that correlates with behavior. Furthermore, for any single region showing anatomy–behavior correlations (e.g., for region volume relative to brain size), there are many possible combinations of: (i) the presence and direction of sex difference in mean volume; ii) the presence and direction of sex differences in behavior; and (iii) the valence of brain–behavior relationships (e.g., “greater volume, greater proficiency” vs. “greater volume, less proficiency”). These possibilities alone represent a vast and complex analytic search space, which increases exponentially if considering multiple brain regions and/or behaviors, and faster still if considering developmental changes rather than cross-sectional variations in anatomy. We outline this theoretical complexity to underline how challenging it would be to test the functional relevance of any observed sex differences in brain anatomy. In the absence of specially tailored study designs to deal with this complexity, there is no empirical basis for arbitrating as to whether a given sex difference in regional brain anatomy may or may not have functional relevance. Prior to initiating research in this field, we suggest that researchers consider whether their personal ethics align with the potential societal implications of this type of work [136, 147].

Careful presentation and framing of findings on sex differences in human brain anatomy—especially with regard to any potential functional implications—may help to limit wider misinterpretations that erroneously deflate or inflate their significance. This caution is especially important given historical precedents for biological data having been misused to justify or explain social inequities [13–15], and the modern risk for discussions of biological sex differences to be unhelpfully simplified, charged, amplified and binarized by social media [148]. Researchers can take proactive steps to decrease the likelihood of such adverse outcomes by explicitly outlining the limits of findings on first publication, including what they do and do not establish, and which hypothesis should be prioritized in follow-up research to replicate and contextualize findings. For example, multiple studies have demonstrated that males exhibit more variability in brain size and structure [116, 117, 123, 149]. In some cases, these results have been misused to justify current gender disparities in STEM fields (due to a higher frequency of males at the “top-end” of neuroanatomical distributions) [150]. However, in other cases, researchers have proactively preempted such biased interpretations by thoughtfully considering the full space of theoretical possibilities and engaging with this space through scientific data-based inquiry. For example: (i) while O’Dea and colleagues [150] confirm that girls exhibit higher average, but less variable grades, they note that by the time girls graduates, they are just as likely as boys to have earned high enough grades to pursue a career in STEM; and (ii) while Wierenga and colleagues [116] confirm greater neuroanatomical variability among men, they note that extreme brain structure (in either direction) may be costly due to energy-induced tradeoffs between volume and conduction time [151].

Following these examples, we argue that any evidence for structural sex differences in the human brain should be discussed from simultaneously scientific and anti-sexist viewpoints. Presenting results on sex-biased brain anatomy from an anti-sexist viewpoint not only requires a mindful avoidance of functional inferences that lack evidence, but also an active effort to highlight the lack of such evidence and carefully define the limits of one's results. Exercising such caution in functional inferences is fully compatible with an impartial consideration of available data on structural differences.

## Conclusions

As detailed above, the best available evidence to date—generated from a direct analysis of tens of thousands of individuals [2, 3] and direct comparisons of results across independent large cohorts [8, 9]—suggests that the human brain shows highly reproducible sex differences in regional brain anatomy above and beyond sex differences in overall brain size. These are differences in the mean value of largely overlapping distributions, and they show small-to-moderate effect sizes; however, we do not understand their microstructural basis, the causal factors shaping them, or if they facilitate sex differences or equivalences in behavior and cognition. There is also a pressing need to better understand the developmental unfolding of sex differences in human brain anatomy over time. This work will benefit from the recent expansion of large longitudinal neuroimaging datasets of human development, as well as recent advances in the methods available to model potentially dynamic sex differences in these datasets [152].

The existence of many open questions, such as those outlined above, does not undo the existence of anatomical sex differences, but instead identifies important topics for future research, along one of the many paths that will hopefully lead us to a richer and more equitable understanding of human diversity [104]. In pursuing these paths, it will be crucial to actively safeguard impartiality and objectivity in our work. Such protections are critical to all domains of scientific inquiry, but they are especially so for studies of sex differences in human neuroanatomy. This call for special care reflects the existence of pervasive gender-biases throughout society, in addition to an undeniable history of cherry-picked biological data being misappropriated and misrepresented to support sexist points of view [13–15]. Modern science is not isolated from this history or from society. In particular, the act of communicating research findings to the public necessarily engages with societal biases. Indeed, scientific organizations and publishers have enacted mechanisms to safeguard against such biases coloring the conduct or presentation of research [153, 154]. Therefore, adopting an "anti-sexist" viewpoint (i.e., one that proactively counters sex/gender-based prejudice, discrimination, or expectations guided by stereotyping) helps to insulate the scientific method from potential biases that might otherwise lead towards denials or over-interpretations of research on sex differences in human brain anatomy. Rather than avoiding, dismissing, or over-interpreting findings of brain sex differences, more accurate description could reduce the misrepresentation and misuse of such research, both within the scientific community and throughout society as a whole.

## Supplementary Information

**Additional file 1: Table S1.** Consistencies in the spatial patterning of neuroanatomical sex differences. This table includes regions showing significant (Family Wise Error < 0.05, corrected) GMV sex differences with the same direction in all three contrasts (Fig. 1D, shown in yellow and cyan). These regions are listed in descending order of cluster size. X, Y and Z coordinates indicate the center-of-mass of each region in the MNI space. Anatomical labels were annotated based on the Automated Anatomical Labeling (AAL) atlas [155]. Regions that are below 20 voxels (that is, 0.0675 cm3) are excluded in this table for simplification. L = left; R = right; partition 1/2 = two separate partitions in one region. 

**Additional file 2: Table S2.** Inconsistencies in the spatial patterning of neuroanatomical sex differences. This table includes regions showing significant (Family Wise Error < 0.05, corrected) GMV sex differences with the opposite direction in any two of three contrasts (Fig. 1E). These regions are listed in descending order of cluster size. X, Y and Z coordinates indicate the center-of-mass of each region in the MNI space. Anatomical labels were annotated based on the Automated Anatomical Labeling (AAL) atlas [155]. Regions that are below 20 voxels (that is, 0.0675 cm3) are excluded in this table for simplification. L = left; R = right; partition 1/2 = two separate partitions in one region.

## Data Availability

Not applicable.

## Abbreviations

ADHD: Attention deficit disorder
ASD: Autism spectrum disorder
HCP: Human Connectome Project
OCD: Obsessive compulsive disorder
SHIP: Study of health in Pomerania
sMRI: Structural magnetic resonance imaging
STEM: Science, technology, engineering, math
UKB: UK Biobank
XCI: X chromosome inactivation
