# Supplementary material for: Sex differences in the human brain: a roadmap for more careful analysis and interpretation of a biological reality
Source: Biol Sex Differ. 2022 Jul 26;13:43. doi: 10.1186/s13293-022-00448-w (PMC9327177; doi:10.1186/s13293-022-00448-w)
Supplement: Supplementary file 1 — Additional file 1: Table S1. Consistencies in the spatial patterning of neuroanatomical sex differences. This table includes regions showing significant (Family Wise Error < 0.05, corrected) GMV sex differences with the same direction in all three contrasts (Fig. 1D, shown in yellow and cyan). These regions are listed in descending order of cluster size. X, Y and Z coordinates indicate the center-of-mass of each region in the MNI space. Anatomical labels were annotated based on the Automated Anatomical Labeling (AAL) atlas [155]. Regions that are below 20 voxels (that is, 0.0675 cm3) are excluded in this table for simplification. L = left; R = right; partition 1/2 = two separate partitions in one region. [file 13293_2022_448_MOESM1_ESM.pdf]

Region	Center-of-Mass location (mm in MNI) x	y	z	Volume (cm3)
	**Male > Female**			
Cerebellum_4_5_L	-12.4	-48.5	-17.9	4.867
Cerebellum_4_5_R	18	-45.3	-20.7	4.698
Temporal_Inf_L_partition1	-44	-5.8	-40.8	3.436
Fusiform_R_partition1	31.5	-21.7	-32.2	3.301
ParaHippocampal_R	22.7	-10.3	-29.7	2.518
Cerebellum_6_R	32.2	-47.3	-31.9	2.400
Vermis_4_5	2.5	-55.5	-11.1	2.373
Fusiform_L_partition1	-28.8	-25.9	-30.2	2.234
ParaHippocampal_L	-19.9	-9.2	-30.5	2.140
Lingual_L_partition1	-13.2	-51.6	-8.9	2.096
Putamen_R	26.1	4.1	3	1.931
Temporal_Pole_Mid_L	-45.4	10.6	-35.3	1.681
Temporal_Inf_R_partition1	42.5	-9.2	-41.6	1.374
Cerebellum_6_L	-23.5	-56.8	-28.2	1.235
Vermis_3	2.9	-44.8	-14.5	1.195
Putamen_L	-24.7	-0.5	3.4	1.026
Cerebellum_Crus1_R	37.9	-50.6	-34.5	0.921
Lingual_R_partition1	13.9	-50.2	-9.8	0.739
Cerebellum_3_R	13.1	-38.6	-21.5	0.699
Temporal_Mid_L	-53.9	3	-30.2	0.678
Temporal_Inf_L_partition2	-52.3	-58.4	-20.9	0.604
Hippocampus_L	-24.6	-11.2	-25.4	0.415
Occipital_Inf_L	-46.9	-68.2	-17.8	0.408
Lingual_L_partition2	-10.5	-88.5	-18.5	0.381
Temporal_Pole_Sup_L	-48.2	8.6	-26.6	0.327
Cerebellum_Crus1_L	-42.6	-70.1	-22.9	0.263
Fusiform_L_partition2	-41.9	-69.4	-19.7	0.250
Amygdala_L	-23.3	-3	-26.1	0.246
Cerebellum_3_L	-8.9	-39.7	-19	0.196

Cerebellum_8_L_partition1	-29.6	-54.3	-48.1	0.192
Lingual_R_partition2	10.8	-87.8	-14.1	0.189
Cerebellum_9_L	-15.1	-50.4	-54.6	0.179
Temporal_Inf_R_partition2	51.3	-58.2	-20.9	0.179
Precuneus_L	-9.8	-48.4	2.8	0.172
Calcarine_L	-4.6	-91.2	-15.5	0.165
Hippocampus_R	30.7	-13.3	-27.3	0.162
Cerebellum_8_R	22.4	-64.9	-44.1	0.128
Amygdala_R	25.8	-0.8	-25.3	0.122
Temporal_Pole_Mid_R	25.1	-0.5	-37.1	0.111
Cerebellum_8_L_partition2	-17.5	-51	-59	0.108
Fusiform_R_partition2	43.9	-67.2	-19.7	0.078
	**Female > Male**			
Cingulum_Ant_L	-4.5	39.5	7.2	4.958
Cingulum_Ant_R	7.5	38.8	12.4	4.455
Frontal_Inf_Tri_L	-43.1	28.6	19.1	3.517
Frontal_Medial_Orb_R	5.2	40.6	-10.5	3.311
Frontal_Inf_Tri_R	44.3	29.9	17.9	2.994
Cingulum_Mid_R	6.9	19.8	34.9	2.744
Occipital_Mid_L	-28.9	-76.7	25.3	2.103
Frontal_Medial_Orb_L	-5.2	40	-12.7	1.981
Parietal_Inf_L_partition1	-31.9	-57.1	41.1	1.772
Rectus_R	4.8	35.9	-20.5	1.758
Frontal_Mid_R_partition1	42.1	42	12	1.677
Insula_R	37.5	-18.2	7.4	1.647
Rectus_L	-4.2	34.4	-20.3	1.569
Rolandic_Oper_R	45.5	-23.5	18.5	1.519
Parietal_Inf_R	36.2	-42.7	44.4	1.448
Cingulum_Mid_L_partition1	-0.8	12.5	38	1.232
Frontal_Sup_Medial_L	-7.6	41.1	16.7	1.151
Frontal_Mid_L	-43.8	29.4	24.7	1.083

Postcentral_R	41.7	-26.6	42	1.046
Occipital_Sup_L	-24.2	-77.7	26.3	1.040
Temporal_Sup_R_partition1	48.7	-23.5	8.9	0.965
Frontal_Inf_Orb_R_partition1	23.7	25.2	-19	0.891
Insula_L	-38	-17.1	5	0.854
Frontal_Sup_Medial_R	8	47.9	2.1	0.813
Parietal_Sup_L	-26.2	-63.4	40.6	0.776
Angular_R	35.1	-55.6	42.6	0.692
SupraMarginal_R_partition1	55.8	-23.7	22.1	0.655
Frontal_Sup_Orb_R	18.3	26.7	-19.2	0.628
Frontal_Inf_Orb_L	-21.9	23.5	-18.2	0.601
Frontal_Inf_Oper_R	42.6	18.1	28.7	0.594
Supp_Motor_Area_L	-1.8	-4.5	47.2	0.543
SupraMarginal_R_partition2	41	-32.5	40	0.483
Frontal_Inf_Orb_R_partition2	43.2	40.2	-14.3	0.469
Parietal_Inf_L_partition2	-37.2	-37.9	40.9	0.415
Frontal_Inf_Oper_L	-43	17.7	29	0.378
Supp_Motor_Area_R_partition1	4	-2.5	45.8	0.375
Postcentral_L	-35.9	-36.1	41.7	0.365
Occipital_Mid_R	30.6	-71.9	27.4	0.354
Precuneus_L	-1	-67.1	41.3	0.317
Temporal_Sup_L	-42.2	-17.1	-3.2	0.314
Temporal_Sup_R_partition2	46.9	-37.1	10.7	0.270
Heschl_R	40.5	-20	6.2	0.263
Frontal_Mid_Orb_R_partition1	24	35	-18.6	0.203
Frontal_Mid_Orb_L	-20.4	29.2	-19.1	0.192
Thalamus_L	-3.8	-11.4	8.9	0.179
Caudate_R	9.3	16.6	5.3	0.176
Parietal_Sup_R	31.7	-46.2	44.3	0.169
Frontal_Mid_Orb_R_partition2	31.6	52.9	-4.3	0.162
Cingulum_Mid_L_partition2	-11.5	-37.2	43.9	0.118

Frontal_Mid_R_partition2	32.4	52.2	-1.5	0.078
Supp_Motor_Area_R_partition2	10.7	0.5	48	0.071
