# Supplementary material for: Sex differences in the human brain: a roadmap for more careful analysis and interpretation of a biological reality
Source: Biol Sex Differ. 2022 Jul 26;13:43. doi: 10.1186/s13293-022-00448-w (PMC9327177; doi:10.1186/s13293-022-00448-w)
Supplement: Supplementary file 2 — Additional file 2: Table S2. Inconsistencies in the spatial patterning of neuroanatomical sex differences. This table includes regions showing significant (Family Wise Error < 0.05, corrected) GMV sex differences with the opposite direction in any two of three contrasts (Fig. 1E). These regions are listed in descending order of cluster size. X, Y and Z coordinates indicate the center-of-mass of each region in the MNI space. Anatomical labels were annotated based on the Automated Anatomical Labeling (AAL) atlas [155]. Regions that are below 20 voxels (that is, 0.0675 cm3) are excluded in this table for simplification. L = left; R = right; partition 1/2 = two separate partitions in one region. [file 13293_2022_448_MOESM2_ESM.pdf]

Center-of-Mass location (mm in MNI)

Region	x	y	z	Volume (cm3)
Occipital_Mid_L	-31.3	-93.4	0.2	2.136
Occipital_Mid_R	35.9	-89.1	4.3	1.198
Cerebellum_Crus2_R_partition1	37	-69.9	-41.5	1.002
Cerebellum_8_R	20	-73.7	-52.9	0.577
Frontal_Sup_R	29.8	0.1	62.3	0.543
Cerebellum_Crus1_R	33.6	-71.6	-37.7	0.513
Thalamus_R	7.2	-23.7	4.1	0.476
Cerebellum_Crus2_L_partition1	-29.7	-74.2	-40.5	0.395
Cerebellum_7b_R	17.9	-78.2	-50.3	0.250
Occipital_Inf_L	-26.8	-91.6	-8	0.203
Cerebellum_Crus1_L	-27.5	-74	-38.6	0.196
Cerebellum_7b_L	-17.2	-74.6	-49.2	0.176
Calcarine_L	0.5	-87.3	-7.1	0.132
Calcarine_R	22.3	-93.4	-1.2	0.132
Cerebellum_Crus2_L_partition2	-16.5	-79.1	-49	0.095
Temporal_Pole_Sup_L	-35.6	17.6	-28.4	0.088
Precentral_R	28	-10.2	64.2	0.081
Cerebellum_Crus2_R_partition2	15.5	-80.8	-48.7	0.074
